# Drug–drug–gene interactions and adverse drug reactions

**DOI:** 10.1038/s41397-019-0122-0

**Published:** 2019-12-03

**Authors:** Mustafa Adnan Malki, Ewan Robert Pearson

**Affiliations:** Population Health & Genomics, School of Medicine, University of Dundee, Dundee, UK

**Keywords:** Clinical pharmacology, Pharmacokinetics, Pharmacogenetics

## Abstract

The economic and health burden caused by adverse drug reactions has increased dramatically in the last few years. This is likely to be mediated by increasing polypharmacy, which increases the likelihood for drug–drug interactions. Tools utilized by healthcare practitioners to flag potential adverse drug reactions secondary to drug–drug interactions ignore individual genetic variation, which has the potential to markedly alter the severity of these interactions. To date there have been limited published studies on impact of genetic variation on drug–drug interactions. In this review, we establish a detailed classification for pharmacokinetic drug–drug–gene interactions, and give examples from the literature that support this approach. The increasing availability of real-world drug outcome data linked to genetic bioresources is likely to enable the discovery of previously unrecognized, clinically important drug–drug–gene interactions.

## Introduction

It was previously and alarmingly reported that adverse drug reactions (ADRs) represent the fourth leading cause of death in the USA [1]. A recent review (2015) showed that 3.6% of patients were admitted to hospitals in Europe due to ADRs and 10% of patients developed side effects during their in-patient stay [2]. The latest report issued by MiDatabank in cooperation with the Medicines and Healthcare Products Regulatory Agency, shows an increasing trend in the number of reported ADRs in the period between 2011 and 2016 across the UK [3]. It has also been estimated that ADRs alone cost the NHS £770 M annually [4]. Nonsteroidal antiinflammatory drugs, diuretics, anticoagulants, and antiplatelets have been recognized to be the major culprits, with prescribing errors being major contributors to medication-related adverse events [5]. The chance of these errors increases when patients undergo multiple treatments; a situation that is highly prevalent in elderly patients [6]. There are a number of factors that influence the occurrence of ADRs secondary to drug–drug interactions, such as age, renal function, and other comorbidities. In addition, genetic variation is likely to play a crucial role in the development of ADRs. For example, when only considering genetic polymorphisms in three drug metabolizing enzymes (cytochrome P450 2C9 (CYP2C9), CYP2C19, and CYP2D6), 15% of the ADRs were due to drug–gene interactions, and 19% were due to drug–drug–gene interactions [7]. Incorporation of these gene variants increased the number of predicted clinically critical drug interactions by ~51% [7]. Given the large number of genes involved in drug metabolism and transport, we cannot underestimate the importance of genetic variation in contributing to potential for clinically critical ADRs.

Following the recent advances in pharmacogenomics, the traditional view of drug–drug interactions needs to be modified to include genetic variation. To date the literature on drug–drug–gene interactions (DDGIs) is limited, with only one previous review evaluating the impact of CYP2C9, C19, and 2D6 variants [8]. In this review, we attempt to provide an in-depth framework for the classification of pharmacokinetic DDGIs caused by different mechanisms, and their potential impact to increase clinically critical drug interactions in the context of the polypharmacy seen in modern medicine today.

## Drug–drug–gene interactions

DDGIs can be divided into three main categories: inhibitory interactions, induction interactions, and phenoconversion interactions. Inhibitory and induction interactions can be defined as any interactions that affect the victim drug’s pharmacokinetics (PK) to increase or reduce concentrations of the drug, respectively. Induction or inhibition can occur either with the administration of a perpetrator drug that alters the victim drug metabolism or transport, or with the presence of loss- or gain-of-function (LOF or GOF) genetic variants that alter function of enzymes that alter metabolism or transport of the victim drug, or the combination of both. A DDGI can be thought of as a double hit—whereby the genetic variant and the perpetrator drug combine to act on transporter or metabolism pathways to greatly alter drug concentrations. It is also possible to see phenoconversion—where the interacting drug effect and the genotype have opposing effects, resulting in a temporary phenotype shift e.g. neutralizing/reversing the effect of a GOF genotype when an inhibitory drug is prescribed. In this review we describe, with examples, different cases of interactions under each of the above three categories, focusing initially on metabolizing enzymes, before considering drug transporters.

### Drug–drug-metabolizing enzyme gene interactions (DDMEGIs)

#### Inhibitory interactions

Inhibitory effects of drugs and genotype can alter substrate metabolism by both drug and genotype impacting on the same metabolizing enzyme, or on two distinct routes of metabolism.

In general, poor metabolizers are expected to experience the highest substrate drug plasma concentration, compared with other genotypes, when co-treated with inhibitors. For example, co-administration of simvastatin (a CYP2C9 inhibitor) with warfarin (CYP2C9 substrate) has been shown to reduce warfarin dosage requirements in CYP2C9*3 carriers with a greater percentage as compared with noncarriers (29% vs 5% respectively) [9]. A similar conclusion has been reported with celecoxib (Supplementary Table 1) [10]. The inhibitory effect of drug and genotype is not always additive—genetically poor metabolizers may have only limited further enzyme inhibition by administration of an inhibitory drug. For instance, a statistically significant elevation in rabeprazole (a CYP2C19 substrate) plasma levels was observed in both normal metabolizers and heterozygous genotype carriers after treatment with fluvoxamine (a CYP2C19 inhibitor) while no additional clinically significant elevation was detected with poor metabolizers who have already experienced the highest rabeprazole plasma levels [11]. A similar scenario is seen with other examples (Supplementary Table 1) [12–15].

Where a drug is metabolized by two or more CYP enzymes, then inhibition of one of these enzymes alone (by drug or genotype) may have minimal effect, due to redundancy of the pathways. However, if a genotype and interacting drug affect these different routes of metabolism, then the interaction may be very large. For example, it has been observed that for voriconazole (a CYP2C19 and CYP3A4 substrate) bioavailability is increased markedly (~5.6-fold) in patients who have reduced CYP2C19 activity and are administered with atazanavir or ritonavir (potent CYP3A4 inhibitors) [16]. A similar scenario can be noted with other examples (Supplementary Table 1) [17–19].

Prodrugs, on the other hand, require the function of certain CYPs to be therapeutically active, and in these cases the effect is the opposite to that described above. Clopidogrel, for example, is activated by CYP1A2, CYP2B6, CYP3A4, CYP2C9, and CYP2C19 [20]. Carriers of LOF variants in one or more of these genes and co-administered with their inhibitors are at increased risk for treatment resistance. For instance, carriers of CYP2C19*2 and/or *3 alleles who are treated with clopidogrel and proton pump inhibitors (CYP2C19 inhibitors) were observed to be more likely to have reduced clopidogrel efficacy; the addition of a third risk factor (e.g., calcium channel blockers (CYP3A4 inhibitors)) was also correlated with a greater reduction in efficacy of clopidogrel [21, 22].

**Fig. 1:**
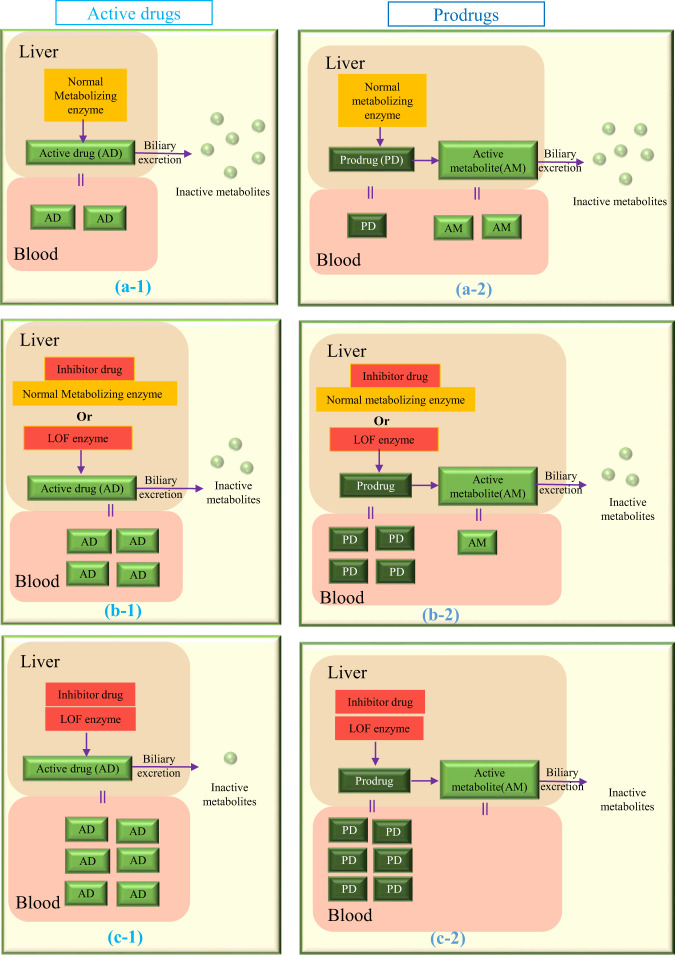
The predicted active drug/active metabolites of prodrugs plasma levels and biliary excretion changes without or with the presence of inhibitors or LOF variants or both on metabolizing enzymes. The predicted active drug/active metabolites of prodrugs plasma levels and biliary excretion changes without (**a-1/a-2**) or with the presence of inhibitors or LOF variants (**b-1/b-2**) or both (**c-1/c-2**) on metabolizing enzymes. **a-1/a-2** represent the normal scenario with no interacting drug or genetic variant. In **b-1/b-2** either an inhibitory drug or loss-of-function variant (LOF) in the metabolizing enzyme, results in reduced metabolism to inactive metabolites, and increased (**b-1**)/decreased (**b-2**) active drug in the systemic circulation. In **c-1/c-2** the presence of inhibitory drug and the LOF genetic variant combine to produce greater increase (**c-1**)/decrease (**c-2**) in the systemic concentration of active drug

Figure 1 shows the predicted changes of plasma levels of active drugs and active metabolites of prodrugs with and without the presence of inhibitors and/or LOF variants.

#### Induction interactions

Increased metabolism of active drugs by an enzyme inducer or GOF variant will result in reduced efficacy of the victim drug. For example, when voriconazole (a CYP2C19 substrate) is co-prescribed with carbamazepine (CYP2C19 inducer) the voriconazole dose is usually increased to overcome this increased metabolism. In a case report, therapeutic concentrations of voriconazole were not achieved, as the patient carried two GOF CYP2C19 *17 variants [23].

The opposite effect is seen with prodrugs. Increased metabolism by an enzyme inducing drug or GOF variant, will result in high plasma levels of active metabolites leading to increased side effects and/or efficacy. Thus, patients carrying CYP2C19*17 GOF variants have increased conversion of clopidogrel to active metabolites resulting in reduced cardiovascular events and/or increased bleeding episodes [24–33]. Co-administration of an inducer of CYP1A2, CYP2C9, and/or CYP3A4 would be expected to result in greater efficacy of clopidogrel, with increased risk of bleeding, however no studies have been published to establish this.

**Fig. 2:**
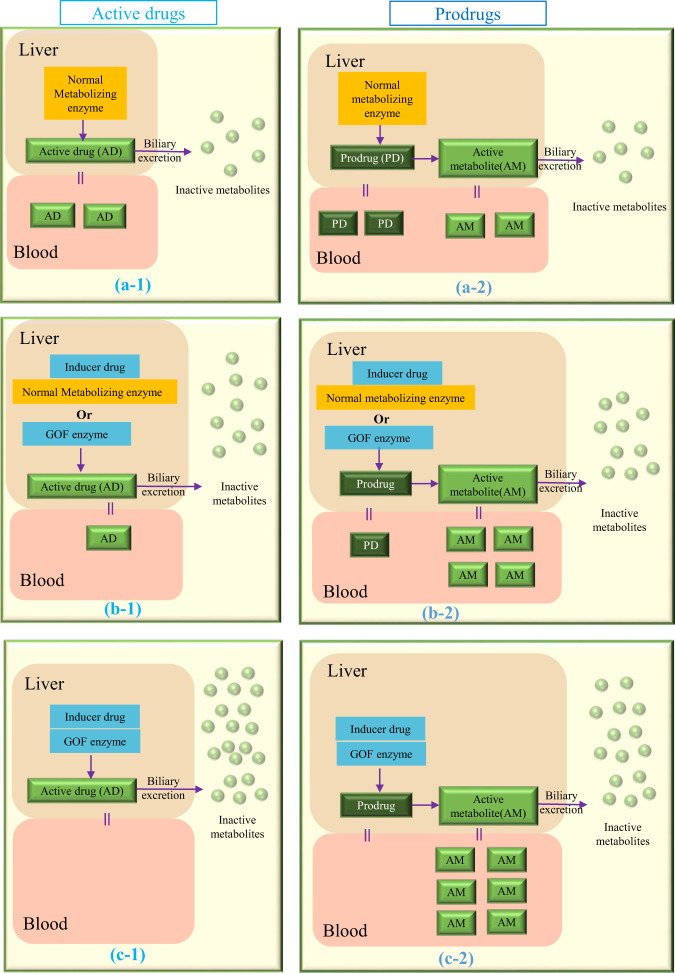
The predicted active drug/active metabolites of prodrugs plasma levels and biliary excretion changes with out or with the presence of inducers or GOF variants or both on metabolizing enzymes. The predicted active drug/active metabolites of prodrugs plasma levels and biliary excretion changes without (**a-1/a-2**) or with the presence of inducers or GOF variants (**b-1/b-2**) or both (**c-1/c-2**) on metabolizing enzymes. **a-1/a-2** represent the normal scenario with no interacting drug or genetic variant. In **b-1/b-2** either an inducer drug or gain-of-function variant (GOF) in the metabolizing enzyme, results in increased metabolism to inactive metabolites, and decreased (**b-1**)/increased (**b-2**) active drug in the systemic circulation. In **c-1/c-2** the presence of inducer drug and the GOF genetic variant combine to produce greater decrease**(c-1)**/increase**(c-2)** in the systemic concentration of active drug

Figure 2 shows the predicted changes of plasma levels of active drugs and active metabolites of prodrugs with and without the presence of inducers and/or GOF variants.

#### Phenoconversion interactions

As described above, a temporary phenotype shift can be seen when the perpetrator drug and genetic effect are opposed. For example, the presence of reduced function CYP2C9 variants results in reduced tolbutamide (a CYP2C9 substrate) metabolism, yet co-treatment with rifampicin (a CYP2C9 inducer) in these patients reverses this genetic effect resulting in a twofold increase in tolbutamide clearance [34]. Conversely, proton pump inhibitors (CYP2C19 inhibitors) treatment with clopidogrel results in phenoconversion in genetically determined ultra-rapid phenotype to a poor metabolizer status indicated by loss of clopidogrel efficacy [35].

The beneficial side of phenoconversion interactions is that genetically determined phenotypes can be normalized by the addition of medications of opposite effects on metabolism. For example, resistance to nortriptyline (CYP2D6 substrate) due to abnormally rapid metabolism has been successfully reversed and normalized with the addition of paroxetine a (CYP2D6 inhibitor), which produces a recovery of nortriptyline therapeutic plasma levels [36].

**Fig. 3:**
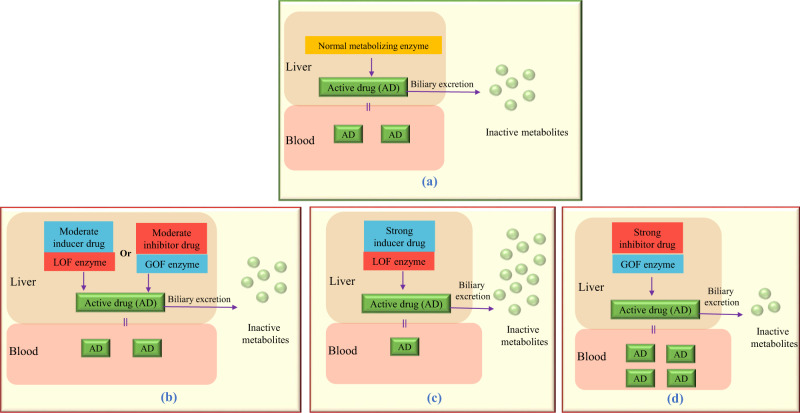
Different scenarios of phenoconversion interactions where genetic effects may be reversed or shifted in the opposite direction. **a** Represents the normal scenario with no interacting drug or genetic variant. In **b** the effect of loss-of-function variant (LOF) or gain-of-function variant (GOF) is reversed with the presence of a moderate inducer drug or a moderate inhibitor drug respectively and results in a clinical outcome similar to the normal situation (**a**). In **c** the presence of a strong inducer drug has temporarily shifted a poor metabolism status into a rapid metabolism status and results in decreased active drug in the systemic circulation. In **d** the presence of a strong inhibitor drug has temporarily shifted a rapid metabolism status into a poor metabolism status and results in increased active drug in the systemic circulation

Figure 3 presents different scenarios of phenoconversion interactions.

### Drug–drug-transporters genes interactions (DDTGIs)

**Fig. 4:**
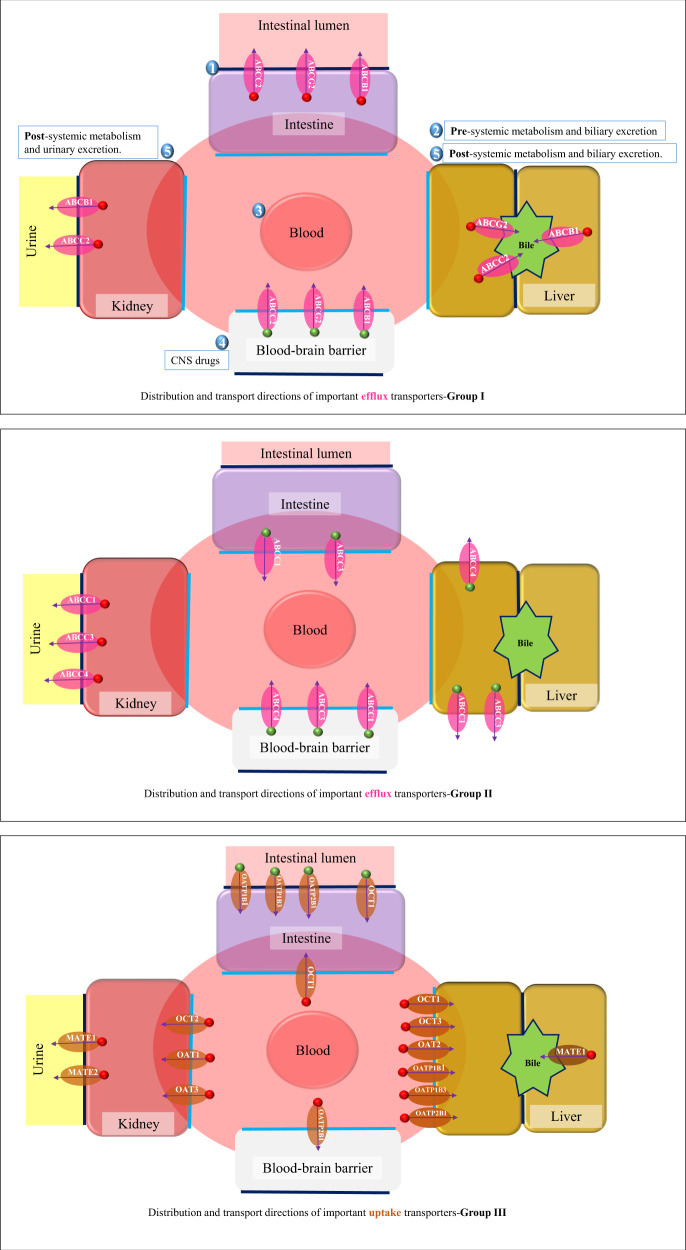
Drug transporters as classified into three categories according to the similarity of the transport directions in different tissue types. Numbers from  to  = order of oral drug movement through different tissue types. Nonoral drug formulations bypass the effect of intestinal transporters. / = increased/decreased substrate drug plasma level is predicted as a result of impairment of this transporter due to LOF variants or inhibitors. The reverse is predicted with GOF variants or inducers. The presence of the two factors (i.e. LOF variant + an inhibitor or GOF variant + an inducer) is predicted to double the clinical impact with neutralizing or shifting the clinical effect when the preparator drug and genetic effect are opposed (phenoconversion interactions).  = Apical membrane.   = Basolateral membrane

Drug transporters govern the movement of pharmaceutical compounds from and into different body tissues. The liver, kidney, blood–brain barrier (BBB), and intestine are the key sites of transporters that influence drug PK. In addition to summarizing the distribution and localization of transporters, Fig. 4 also classifies transporters into three categories according to the similarity of transport directions in different tissue types (the figure has been formulated with the aid of reference [37]). Drug–drug–gene interactions for transporters are less well studied than for metabolizing enzymes. For each subgroup, Drug Transporter-gene interaction (DTGI) studies will be utilized (if no direct DDTGI studies are available) to illustrate each mechanism for potential interaction. Similar to the drug metabolizing enzyme scenarios outlined above, we predict that these interactions may be intensified or reversed, via inhibitory/induction or phenoconversion pathways, with the co-administration of inhibitors or inducers.

#### Efflux transporters

Efflux transporters have been classified into two groups (group I and group II) according to the similarity in the transport directions.

##### Group I

P-glycoprotein 1 (P-gp, ABCB1), multidrug resistance-associated protein 2 (MRP2, ABCC2), and breast cancer resistant protein (BCRP, ABCG2) transporters are expressed in the intestine, liver, kidney, and BBB, sharing similar transport pathways. They efflux substrates back to intestinal lumen, facilitate hepatic and renal excretion (excluding BCRP), and work inversely in the BBB where they protect the brain from the entry of xenobiotics and return them back to systemic circulation. Blocking their function in the intestine, liver, or kidney is expected to elevate a substrate’s systemic exposure (although opposite effects would be predicted if inhibiting transport across the BBB).

In this group, the most evidence for DDTGI comes from drugs altering ABCB1 (P-gp) transport and genetic variants in the gene encoding this transporter. For example, cyclosporine is an ABCB1 substrate. Diltiazem (a moderate ABCB1 inhibitor [38]) has been shown to increase cyclosporin trough concentrations in Chinese patients who carry the TT genotype (low P-gp activity) at rs1045642(C>T) in ABCB1; yet no effect was seen in other ABCB1 genotypes (e.g., CC at rs1045642) [39]. Methadone is also a P-gp substrate, acting in the brain and effluxed across the BBB via P-gp. Patients with the TT genotype at rs1045642 and treated with quetiapine (ABCB1 inhibitor) experienced the lowest increase in methadone plasma levels compared with those with CT or CC genotypes (3% vs 23% vs 33% respectively) [40]. Low methadone plasma levels in this study would be explained by loss of the ABCB1 protective function in the BBB which results in increased intracerebral concentration of this central nervous system (CNS) drug. As a result of a similar DDTGI mechanism, the CNS drug granisetron was associated with increased efficacy in Japanese subjects (Supplementary Table 1) [41].

In some cases, it seems that adding strong inhibitors abolishes the effect of genotype. For example, no additional inhibitory effects were detected in carriers of different genotypes of the rs1045642 (C>T) ABCB1 variant who were either on dabigatran/rivaroxaban-clarithromycin combination or tacrolimus-itraconazole combination (ABCB1 substrates-ABCB1 strong inhibitors [38]) [42, 43].

ABCC2 and ABCG2 would be predicted to follow similar interaction scenarios as ABCB1, yet we were unable to find any studies that report DDTGIs for these transporters.

##### Group II

Unlike group I transporters, there are no published studies describing DDTGIs for group II transporters. So here we report DGTIs to highlight the potential mechanisms whereby genes and drugs that alter these transporters may influence drug outcomes. MRP1(ABCC1), MRP3 (ABCC3), and MRP4(ABCC4) share the similar transport direction in the kidney and BBB as the Group I transporters. However, in the liver, they are expressed in the basolateral membrane working to pump drugs back into systemic circulation. MRP1, for example, transports the active metabolite of irinotecan (SN-38) out of hepatocytes into the blood contributing to the well-known side effect of irinotecan induced neutropenia [44]. The reduced function variant, rs17501331, in the *ABCC1* gene is associated with low incidence of neutropenia; the reverse effect was detected with the GOF variant rs6498588 in the same gene [45]. In some cases, increased activity of the MRP1 transporter can be advantageous, as seen with methotrexate hepatotoxicity where carriers of wild-type genotype of *ABCC1* rs246240 (A>G) variant are at higher risk for developing methotrexate toxicity compared with carriers of reduced function alleles [46]. Of note, MRP1 is also expressed in the myocardium protecting the heart from the entry of xenobiotics [47]. For example, the reduced transport associated with the rs45511401 (G>T) in ABCC1 increases the chance of developing cardiotoxicity due to intracellular accumulation of doxorubicin [48]. MRP1 and MRP3, in contrast to P-gp, MRP2 and BCRP, are expressed in the basolateral membrane of the intestine effluxing substrates into the portal circulation. As orally administered drugs are first exposed to intestinal transporters, any modification of their role might affect drug concentration in the other tissues (liver, kidney, or BBB). C.1037 C>T and c.1820G>A ABCC3 variants, for example, have low transport activity [49] suggesting their potential to diminish the bioavailability of oral MRP3 substrates irrespective of subsequent alteration in transport into other tissues, or subsequent metabolism.

#### Uptake Transporters (Group III)

In the liver, kidney, and BBB, all important uptake transporters (organic cation transporters (OCTs)1/2/3, organic anion-transporting polypeptide (OATP) 1B1/1B3/2B1, and multidrug and toxic compound extrusion proteins (MATE) 1/2), follow an identical main route for transporting their substrates: from systemic circulation into different tissues or urine/bile in case of MATEs. Consequently, reducing or increasing these transport capacities would result in increased or reduced systemic drug concentrations respectively. The reverse effects are seen with the uptake transporters expressed in the intestinal apical membrane such as OATPs and OCT1 since the transportation pathway is in the opposite direction.

In some circumstances, altering uptake transporter function can increase ADRs. For example, it has been observed that carriers of two OCT1 (*SLC22A1*) reduced function alleles who were treated with OCT1 inhibitors were over four times more likely to develop gastrointestinal side effects with metformin (an OCT1 substrate) treatment, which would be attributable to metformin accumulation in the intestinal lumen (assuming apical OCT1 localization) [50]. This finding was supported by a previous study [51]. At the level of renal uptake transporters, other DDTGIs have been reported in which carrying the mutant alleles and the co-administration of inhibitors was linked to increased metformin plasma levels/toxicity or reduced clearance (see Supplementary Table 1) [52, 53]. By contrast, reducing transport in some cases may reduce certain side effects. For instance, cisplatin (a OCT2 (*SLC22A2*) substrate) is both a nephrotoxic and an ototoxic agent. People carrying the rs316019 (C>A) OCT2 mutation were protected from these adverse reactions as the variant resulted in reduced transport of cisplatin into the kidney and the inner ear (cochlea) (where OCT2 is expressed as well) [54–56].

In many situations, the efficacy of a drug relies upon the ability of that drug to access certain tissues. Statins are taken up into the liver by OATP1B1(*SLCO1B1*) and this is crucial for their lipid lowering effect. Reducing this uptake pathway reduces statin efficacy and raises plasma concentrations, resulting in myopathy and, rarely, rhabdomyolysis. The rs4149056 (T>C) (SLCO1B1*15) variant has been widely studied, and in 23 studies [57–79], this variant has been persistently connected to increased statin plasma exposure, muscle aches, dose reduction, and/or treatment-resistant phenotypes. A number of other DDGIs have been described for the SLCO1B1 transporter. For example, although the increase in pravastatin (SLCO1B1 substrate) AUC after treatment with ritonavir (SLCO1B1 inhibitor) was not statistically significant (21% increase vs pravastatin alone) a large interaction was seen in those carrying the SLCO1B1*15 or *17 haplotypes, with a resulting 113% elevation in pravastatin AUC [80]. Other DDTGIs with the similar mechanism have also been published (see Supplementary Table 1) [81–83]. Interestingly, unlike the ritonavir example just outlined, in some situations reduced function variants do not show any significant PK change until after the addition of inhibitors. For example, patients with AG or AA genotypes at rs2289669(G>A) of the MATE1 transporter only had significantly lower metformin (MATE1 substrate) clearance compared with carriers of GG genotype after treatment with ranitidine (a MATE1 inhibitor) [84].

## DDGIs and challenges in clinical practice

Metabolizing enzyme and transporter substrates, inducers, or inhibitors are not fully documented in many popular drug interaction databases, leaving physicians unaware of potentially important interactions. In addition, most of the resources commonly used by prescribers (e.g., Stockley’s, Micromedex, Drug.com, RxList, or other drug interaction checkers) do not consider genetic variation when classifying drug interactions into minor, moderate, or major classes. Genetic variation may markedly increase or ameliorate the severity of potential drug interactions and do need to be considered when considering real-world use of drugs.

This review has discussed the different mechanisms of interactions in their simplest forms with the assumption that the patient is free of transporter polymorphisms or inhibitors/inducers in the case of discussing DDMEGIs and vice versa with DDTGIs. However, in real-world clinical practice, achieving precisely tailored drug therapy requires a detailed examination of all mutations in the candidate enzyme or transporter genes with good awareness of the entire prescribed medications and possible pathways of interaction. Thus, the clinical scenario ranges from a relatively simple picture where the effect of genotype and interacting drug(s) can be approximated and treatment altered accordingly, to a far more complex scenario where physiologically based PK (PBPK) modeling may be helpful and where evaluation of large scale clinical data linked to genotypes is required to evaluate the clinical impact of multiple interacting drugs/multiple genotypes on drug outcomes.

Consider a relatively simple scenario: a patient with type 2 diabetes treated with metformin (has no effect on CYPs) who carries reduced function variants in *CYP2C9* (*2 or *3 variants) and who is started on gliclazide (CYP2C9/19 substrate). Reduced metabolism of gliclazide will result in increased efficacy [85] and increased risk of hypoglycemia [86]. The metformin use will not alter this DGI. However, if this patient were also treated with pioglitazone and/or atorvastatin (both are CYP2C9/19 inhibitors) they would be at potentially even greater risk of gliclazide-induced hypoglycemia and should be treated with a reduced dose of gliclazide. However, even for this simple scenario, such DDGI studies have not been reported; nor have dosing algorithms been developed to date for patients with CYP2C9 variants prescribed sulphonylureas and as such it is difficult to implement this into drug interaction calculators.

There are many more complex scenarios where, for example, a combination of both metabolizing enzyme and transporter LOF/GOF variants, as well as inhibitors/inducers are included. This kind of interaction may be only initially predictable when all their subinteractions result in the same clinical effect. For instance, reduced CYP3A4 and SLCO1B1 activities can both result in increased AUC of the substrate drug and a greater harm would be anticipated. Carriers of the TC genotype of SLCO1B1 rs4149056 (T>C) variant who are treated with amlodipine (CYP3A4 inhibitor) experienced a 90% increased simvastatin AUC compared with subjects not treated with amlodipine and wild-type for rs4149056 [87]. A similar scenario was reported with other two case reports (see Supplementary Table 1) [88, 89].

In other situations, subinteractions do not share a similar clinical effect. Here, predicting the overall clinical outcome is challenging. As an illustration, oral rosuvastatin is mainly eliminated via biliary excretion with a minor contribution of CYP2C9 to its metabolism [90]. This implies that its transporters (e.g., ABCC2, ABCG2, ABCC1, and SLCO1B1) are the core players in its elimination. The concomitant administration of verapamil (an ABCC1/2 inhibitor) and venlafaxine (an ABCG2 inducer) in those who have inherited CYP2C9*3 and/or SLCO1B1 rs4149056 (T>C) LOF variants results in unpredictable clinical consequences. CYP2C9, SLCO1B1, and ABCC2 impairment would boost rosuvastatin AUC, inducing ABCG2 would lower rosuvastatin AUC, and inhibition of ABCC1 could result in both increase or decrease in AUC (high AUC if the site of interaction is in the kidney and low AUC if it is in the intestine or liver). The exact estimation of the predicted net AUC following a certain DDGI relies on calculating the contribution of each metabolizing enzyme and transporter to the elimination process (i.e. degree of sensitivity of substrates), inhibition/induction potency of the perpetrator agent or the net effect of multiple inhibitors, inducers, or both, and the net percentage of reduction/elevation in the enzyme/s and/or transporter/s activity caused by a single or more SNPs. The outcome of such a hugely complex scenario is impossible to predict by the clinician, and requires a clinical support tool based upon a PK DDGI prediction algorithm. Most of the current work concentrates on generating either DD or DG interaction predictors rather than the combined effect of both drugs and variants. However, using PBPK models, one predictor tool (https://www.ddi-predictor.org/) has recently been successfully generated to estimate drug exposure and the recommended dose following the dual action of both the perpetrator drug and mutations in certain CYPs (CYP2D6, CYP2C9, and CYP2C19) [91]. Other PBPK models do attempt to incorporate genotype and drug–drug interactions, but these do not model transporter variants well and have yet to translate through into clinically useful tools [92].

An alternative method to evaluate the impact of DDGIs is via metabolizing enzymes and transporters endogenous biomarkers rather than plasma concentrations of substrate drugs. Multiple enzymes/transporters-related biomarkers have been identified [93]. For instance, it has been shown that the cholesterol, cortisone, and cortisol metabolites: 4β-hydroxycholestrol,6β-hydroxycortisone, and 6β-hydroxycortisole, respectively, which are catalyzed by CYP3A4 activity, are increased under the effect of inducers and decreased with inhibitors of CYP3A4. It was also recognized that bufotenine is a major metabolite resulting from the metabolizing activity of CYP2D6. With regard of transporters, several studies have observed the association between increased bilirubin plasma levels and reduced hepatic OATP1B1/1B3 uptake function. The similar scenario was noted recently with the novel biomarkers coproporphyrins I and III (CPs I and III) where plasma CPs levels elevated with the inhibition of these transporters to a similar extent as with rosuvastatin. In DDGIs studies, endogenous biomarkers can be utilized to predict the effect of both genetic variants and inhibitors/inducers on the substrate drugs plasma levels.

It is worth noting that potential DDIs do not necessarily reflect actual interactions. It has been observed that clinically significant interactions are consistently lower than theoretically predictable interactions [94]. However, the authors noted that 20% of ADRs are linked with DDIs; most of them are serious with a high percentage of fatal cases. They also concluded that therapeutic failure secondary to DDIs, which is usually underestimated, represents a considerable part of total DDIs-related undesirable effects. The degree of clinical significance can be judged by observing other risk factors associated with a potential DDI such as polypharmacy and genetic variants. Polypharmacy is commonly seen with elderly and hospitalized patients making them the most vulnerable patient’s subgroups to clinically significant interactions besides carriers of risky genetic variants. In addition, not all types of DDGIs are expected to be common. Induction and phenoconversion DDGIs are predicted to be seen with lower incidence compared with inhibitory DDGIs as the majority of perpetrator drugs are inhibitors rather than inducers and most of functional genetic variants are loss rather than GOF mutations.

The increasing availability of ‘big data’ linking health data and genomics has the potential to evaluate the real-world clinical impact of multiple drugs/multiple variant interactions. A number of data sets are now available or about to become available for study. In Scotland national prescribing and linked outcomes are available for the entire population enabling evaluation of real-world DDIs, and with an increasing bioresource (https://www.registerforshare.org) it should be possible to evaluate DDGIs in ~500 K people over the next few years. In addition, other resources such as UK biobank including genetic information on 500 K individuals (with primary care data available on 200 k during 2018) and other national bioresources (such as the Danish biorepository) and US bioresources linked to EHRs (EMERGE network) will enable the evaluation of n-way DDG interactions to identify clinically important interactions that can be incorporated into clinical decision support tools in the future.

## Conclusion

Dozens of new pharmaceutical compounds enter the market each year and a considerable number of patients are prescribed multiple drugs that necessitate the utilization of drug interaction databases for better management. One of the major limitations of these drug interaction checkers is the omission of the genetic effect on drug interactions. This reflects both the lack of clinical studies that quantity potential DDGIs and the fact that genetic information is rarely available on patients at the point of prescribing. This review has illustrated, with some examples, various mechanisms by which DDGIs can occur at the level of metabolizing enzymes, drug transporters, or both (this has been summarized in Supplementary Table 1). We have also shown the different degrees of complexity clinicians may face in judging the predicted clinical outcome following a certain DDGI. The more factors that are included, the more challenging it becomes to evaluate the outcome. There is a need for PBPK models, clinical studies and real-world evaluation of drug outcomes linked to genetic information to develop clinical useful DDGI models, to reduce adverse DDIs and improve drug outcomes in the setting of increasing multi-morbidity and polypharmacy.

## Supplementary information

Supplementary Table 1
