# Supplementary material for: Drug–drug–gene interactions and adverse drug reactions
Source: Pharmacogenomics J. 2019 Dec 3;20(3):355–66. doi: 10.1038/s41397-019-0122-0 (PMC7253354; doi:10.1038/s41397-019-0122-0)
Supplement: Supplementary file 1 — Supplementary Table 1 [file 41397_2019_122_MOESM1_ESM.docx]

| Metabolizing enzymes interactions  Table 1: classification of drug-drug-gene interactions according to their different mechanisms as observed from findings of clinical studies or case reports. | | | |
| --- | --- | --- | --- |
| Inhibitory interactions-the victim drug as an active drug | | | |
| Clinical outcome | Example/s | Interaction model | Interaction type |
| CYP2C9*3 carriers require lower warfarin dose compared to non-carriers [9]. | Warfarin+simvastatin+CYP2C9*3 in CYP2C9 gene | Substrate + inhibitor +LOF variants in the same enzyme inhibited by the inhibitor | Single enzyme interactions  (Both the inhibitor and the genotype affect the same enzyme) |
| Although the greater increase in the substrate drug plasma level is always seen with poor metabolizers, the effect of adding inhibitors is observed more clearly in normal metabolizers followed by heterozygous genotype carriers with the lowest effect being with poor metabolizers as they already carry reduced activity metabolizing enzymes [11-15]. | Lansoprazole/rabeprazole+ fluvoxamine+ poor metabolism genotype in CYP2C19 gene. |  |  |
|  | Omeprazole+ moclobemide+ poor metabolism genotype in CYP2C19 gene. |  |  |
|  | Metoprolol+ diphenhydramine+ poor metabolism genotype in CYP2D6 gene. |  |  |
|  | Metoprolol+ dronedarone+ poor metabolism genotype in CYP2D6 gene. |  |  |
| Increased bleeding risk with warfarin in those carrying the variant CYP2C9 alleles [10]. | Warfarin + celecoxib+ CYP2C9*2/*3 in CYP2C9 gene | Substrate +2^nd^ substrate (competitive inhibitor) +LOF variants in the same enzyme inhibited by the inhibitor |  |
| Poor metabolizers experienced marked AUC increase from the substrate drugs compared to normal metabolizers [16-19]. | Voriconazole + atazanavir/ritonavir (CYP3A4 inhibitors) + poor metabolism genotype in CYP2C19 gene. | Substrate +inhibitor +LOF variants in an enzyme differ from the one inhibited by the inhibitor | Multiple enzymes interactions  (The inhibitor and the genotype affect different enzymes) |
|  | Tacrolimus + voriconazole (CYP3A4 inhibitor) + poor/intermediate metabolism genotype in CYP2C19 gene. |  |  |
|  | Voriconazole + erythromycin (CYP3A4 inhibitor) + poor metabolism genotype in CYP2C19 gene. |  |  |
|  | Lansoprazole + clarithromycin (CYP3A4 inhibitor)+ poor metabolism genotype in CYP2C19 gene. |  |  |
| Inhibitory interactions-the victim drug as a prodrug | | | |
| Poor metabolizers were more likely to develop clopidogrel resistance compared to normal metabolizers [21]. | Clopidogrel + proton pump inhibitors (CYP2C19 inhibitors) + CYP2C19*2/*3 in CYP2C19 gene | Substrate +inhibitor +LOF variants in the same enzyme inhibited by the inhibitor | Single enzyme interactions |
| Poor metabolizers developed marked reduction in clopidogrel efficacy compared to normal metabolizers [22]. | Clopidogrel + proton pump inhibitors (CYP2C19 inhibitors) + CYP2C19*2/*3 in CYP2C19 gene + calcium channel blockers (CYP3A4 inhibitors) | Substrate + 2 inhibitors of 2 different enzymes +LOF variants in one of the enzymes inhibited by one of the two inhibitors. | Multiple enzymes interactions |
| Induction Interactions | | | |
| Major loss of voriconazole efficacy was seen in one patient [23]. | Voriconazole + carbamazepine+ CYP2C19*17/17  in CYP2C19 gene | Substrate +inducer +GOF* variant in the same enzyme induced by the inducer | Single enzyme interactions |
|  |  |  | Phenoconversion Interactions |
| Loss of clopidogrel efficacy [35]. | a) Ultra-rapid metabolism > poor metabolism    Clopidogrel+ proton pump inhibitors+ CYP2C19*17  in CYP2C19 gene | Substrate +inhibitor+ GOF variant in the same enzyme inhibited by the inhibitor | Single enzyme interactions |
| Normal nortriptyline therapeutic plasma levels [36]. | b) Ultra-rapid metabolism > normal metabolism  Nortriptyline + paroxetine + ultra-rapid metabolism genotypes in CYP2D6 gene. |  |  |
| Twofold increase in tolbutamide clearance [34]. | c) Poor metabolism> ultra-rapid metabolism  Tolbutamide + rifampin+ intermediate or poor metabolism genotypes in CYP2C9 gene | Substrate +inducer+ LOF variant in the same enzyme induced by the inducer |  |
| Transporters interactions | | | |
| Inhibitory interactions-efflux transporters | | | |
| TT genotype carriers at rs1045642 variant show increased cyclosporin trough concentration (no effect was detected with other genotypes) [39] and the lowest increase in methadone plasma level compared to other genotypes [40]. | Cyclosporine + diltiazem + rs1045642(C>T) in ABCB1 gene | Substrate/s +inhibitor/s +LOF* variant in the same transporter inhibited by the inhibitor | Single transporter interactions |
|  | Methadone + quetiapine + rs1045642(C>T) in ABCB1 gene |  |  |
| GG carriers show the highest response from the substrates compared to other genotypes [41]. | [Granisetron + dexamethasone] + [doxorubicin+ cyclophosphamide] + rs2032582(G>T)* in ABCB1 gene  **G, but not T, allele was linked to reduced ABCB1 activity in this study with Japanese population.* |  |  |
| No effect was seen with any genotype  [ 42,43]. | Dabigatran/rivaroxaban + clarithromycin **or**  Tacrolimus + itraconazole  + rs1045642 (C>T) in ABCB1 gene |  |  |
| Inhibitory interactions-uptake transporters | | | |
| Carriers of two LOF alleles were over four times more likely to develop metformin intolerance [50]. | Metformin + OCT1 inhibitors (tricyclic antidepressants, citalopram, proton pump inhibitors, verapamil, diltiazem, doxazosin , spironolactone , clopidogrel , rosiglitazone , quinine , tramadol ,and codeine) + R61C, C88R , G401S, M420del, and G465R in OCT1 gene. | Substrate +inhibitor +LOF variant in the same transporter inhibited by the inhibitor | Single transporter interactions |
| Increased metformin plasma concentrations [52]. | Metformin + trimethoprim (MATE1/OCT2 inhibitor) +  rs2289669 in MATE1 gene **or**  rs316019 in OCT2 gene |  |  |
| TT genotype carriers show the lowest rate of metformin clearance compared to other genotypes [53]. | Metformin + cimetidine + c.808G>T in OCT2 gene. |  |  |
| Carrying the variant alleles has elevated pravastatin AUC by 113%. No significant interaction was seen with normal genotypes. [80]. | Pravastatin + ritonavir + SLCO1B1*15 or *17 haplotypes in SLCO1B1 gene. |  |  |
| CC genotype carriers displayed higher plasma concentrations from the substrate drugs compared to other genotypes [81,82]. | Olmesartan + pravastatin **or**  Repaglinide + gemfibrozil  + rs4149056 (T>C) in SLCO1B1 gene. |  |  |
| Although no significant difference in atorvastatin plasma concentration between the three genotypes was detected, a clear increase can be observed with CC genotype carriers and on the drug combination compared to those on atorvastatin only [83]. | Atorvastatin + rifampicin + rs4149056 (T>C) in SLCO1B1 gene. |  |  |
| AG and AA genotype carriers experienced lower metformin clearance compared to GG genotype carriers [84]. | Metformin + ranitidine + rs2289669(G>A) in MATE1 gene |  |  |
| Enzyme/s + transporter/s interactions | | | |
|  |  | Substrate + transporter/s and/or enzyme/s inhibitor/s and/ or inducers and/or 2nd substrate + LOF/GOF variants in one enzyme or more and/or in one transporter or more | Multiple enzyme/s and transporter/s interactions  (The perpetrator drug and the genotype affect both enzyme/s and transporter/s of the same substrate) |
| Simple (predictable) interactions (sub-interactions result in the same clinical outcome) | | | |
| Subjects with TC genotype experienced a 90% increase in simvastatin AUC compared to those not treated with amlodipine and carry normal SLCO1B1 genotype [87]. | Simvastatin + amlodipine (CYP3A4 inhibitor) + rs4149056 (T>C,LOF) in SLCO1B1 gene. |  | |
| An elevation in creatine kinase level was observed in a patient on this drug-drug-genotypes combination [88]. | Fluvastatin + telmisartan (ABCC2 inhibitor) + CYP2C9*3 and -24C > T (LOF variants) in CYP2C9 and ABCC2 genes respectively. |  |  |
| A patient on this drug-drug-genotypes combination has developed rhabdomyolysis and acute renal failure [89]. | Atorvastatin + pantoprazole (SLCO1B1 and ABCB1 inhibitor, and CYP3A4 substrate) + CC genotype* at rs4149056 (T>C) in SLCO1B1 gene + TT genotype* at rs1045642 (C>T) in ABCB1 gene.  ** CC and TT genotypes are associated with reduced activity.* |  |  |
| Complex (unpredictable) interactions (sub-interactions result in different clinical outcomes) | | | |
| Advanced PBPK prediction tools are required to be developed with conducting real-world clinical studies to address and understand this kind of complex interactions. | Rosuvastatin + verapamil (ABCC1/2 inhibitor) and venlafaxine (ABCG2 inducer) + CYP2C9*3 and/or rs4149056 (T>C) LOF variants in CYP2C9 and/or SLCO1B1 genes respectively.  *This example has been used for illustration purpose only (no studies were reported).* |  | |

* LOF = Loss of function

* GOF = Gain of function
